# MEAS: memory encryption and authentication secure against side-channel attacks

**DOI:** 10.1007/s13389-018-0180-2

**Published:** 2018-01-25

**Authors:** Thomas Unterluggauer, Mario Werner, Stefan Mangard

**Affiliations:** Institute for Applied Information Processing and Communications, Graz University of Technology, Inffeldgasse 16a, 8010 Graz, Austria

**Keywords:** Side-channel attacks, DPA, Memory, Encryption, Authentication

## Abstract

Memory encryption is used in many devices to protect memory content from attackers with physical access to a device. However, many current memory encryption schemes can be broken using differential power analysis (DPA). In this work, we present Meas—the first Memory Encryption and Authentication Scheme providing security against DPA attacks. The scheme combines ideas from fresh re-keying and authentication trees by storing encryption keys in a tree structure to thwart first-order DPA without the need for DPA-protected cryptographic primitives. Therefore, the design strictly limits the use of every key to encrypt at most two different plaintext values. Meas prevents higher-order DPA without changes to the cipher implementation by using masking of the plaintext values. Meas is applicable to all kinds of memory, e.g., NVM and RAM. For RAM, we give two concrete Meas instances based on the lightweight primitives Ascon, PRINCE, and QARMA. We implement and evaluate both instances on a Zynq XC7Z020 FPGA showing that Meas has memory and performance overhead comparable to existing memory authentication techniques without DPA protection.

## Introduction

Memory encryption is the standard technique to protect data and code against attackers with physical access to a memory. It is widely deployed in state-of-the-art systems, such as in iOS [2], Android [22], Mac OS X [1], Windows [19], and Linux [26, 36]. Typical encryption schemes employed in these systems are Cipher-Block-Chaining with Encrypted Salt-Sector IV (CBC-ESSIV) [20], Xor-Encrypt-Xor (XEX) [47], and XEX-based Tweaked codebook mode with ciphertext Stealing (XTS) [30]. These schemes successfully prevent attackers from accessing memory content when the device is shut off and the encryption key is not present on the device, e.g., an encrypted USB flash drive.

Contrary to that, in many situations in the Internet of Things (IoT), a physical attacker is in possession of a running device, or can turn a device on. In these cases, the attacker can, for example, observe and tamper with data in RAM. As a result, memory encryption and tree-based authentication techniques, e.g., Merkle trees [40], Parallelizable Authentication Trees [27] (PAT) and Tamper Evident Counter [18] (TEC) trees, are increasingly deployed to protect data in RAM. As one prominent example, RAM encryption and authentication was only recently adopted in consumer products with Intel SGX [25]. Similarly, there are efforts to encrypt RAM on AMD [32] and ARM systems [29] as well.

However, whenever a physical attacker has access to a running device, the attacker is also capable of performing side-channel attacks. This means that the attacker cannot just read and tamper with the memory, but is also capable of measuring side-channel information, such as the power consumption of the hardware, during the encryption and authentication of the memory. The attacker can then exploit such side-channel information to learn the secret key used for memory encryption and authentication. In practice, an attacker performing both passive, e.g., bus probing, and active, e.g., data spoofing, attacks on the memory, is also capable of observing side-channel information, e.g., by attaching an oscilloscope for measuring the power, during the actual encryption or authentication process. As such, side-channel attacks are realistic for any physical attacker when given access to a running device. One particularly strong class of side-channel attacks is differential power analysis [34] (DPA), which allows successful key recovery from observing the power consumption during the encryption/decryption of several different data inputs. DPA attacks effectively accumulate side-channel information about the key being used by observing multiple encryptions/decryptions under the same key.

However, contemporary memory encryption and authentication schemes that protect memory against physical attackers, e.g., [17, 25, 44, 48, 54, 55], lack the consideration of side-channel attacks and DPA in particular. More concretely, the security of contemporary schemes is built upon the assumption of a microchip that is secure against active and passive adversaries and which does not leak any information about the key via side channels. However, as pointed out before, the assumption that side-channel attacks on microchips are infeasible is too strong. In fact, DPA attacks were quite recently shown to pose a serious threat to memory encryption on general-purpose CPUs. While the DPA presented in [57] breaks many contemporary memory encryption schemes, the practical attacks in [5, 37, 50, 57] document the feasibility of DPA on memory encryption and authentication on state-of-the-art systems.

In principle, there exist techniques to protect cryptographic primitives against DPA attacks. For example, an implementation can be protected by changing the hardware such as by applying masking techniques [12, 23], which use randomization to make the side-channel information independent from the actually processed value. However, protecting implementations of cryptographic primitives against DPA is expensive and a tough problem in an active field of research existing for almost two decades. The massive overheads for DPA-protected implementations range between a factor of four and a few hundred [6, 10, 42, 45] and would thus render current memory encryption and authentication schemes in latency sensitive applications impractical. In contrast, more efficient solutions are in sight when considering side-channel protection throughout the cryptographic design and looking for potential synergies.

### Contribution

In this paper, we solve the problem of protecting data in memory against physical attackers in possession of a running device. More concretely, we solve the stringent problem of DPA attacks on memory encryption and authentication without additional memory overhead over conventional schemes.

We approach the topic with a detailed analysis of the security of fresh re-keying [33, 39] as a promising mechanism to prevent DPA on memory encryption. While re-keying completely thwarts DPA on the cryptographic key, our major result here is that re-keying provides merely first-order DPA security for the memory content itself. In particular, we show that the read–modify–write access patterns inevitably occurring in encrypted memory allow for profiled, higher-order DPA attacks that leak constant plaintext data when re-keying is applied to memory encryption.

Second, we build on our analysis and present Meas—the first Memory Encryption and Authentication Scheme secure against DPA attacks. The scheme is suitable for all kinds of memory including random access memory (RAM) and non-volatile memory (NVM). By making use of synergies between fresh re-keying and authentication trees [18, 27, 40], Meas simultaneously offers security against first-order DPA and random access to all memory blocks. In more detail, Meas uses separate keys for each memory block that are stored in a tree structure and changed on every write access in order to strictly limit the use of each key to the encryption of two different plaintexts at most. For higher-order DPA security, Meas performs data masking by splitting the plaintext values into shares and storing the encrypted shares in memory. This allows to flexibly extend DPA protection to higher orders in trade for additional memory. For all DPA protection levels, Meas does not require DPA-protected implementations of the cryptographic primitives, making Meas suitable for common off-the-shelf (COTS) systems equipped with unprotected cryptographic accelerators. However, Meas is also an ideal choice for constructing a DPA-secure system from scratch as engineers do not have to cope with complex DPA protection mechanisms within the cipher implementation.

Third, we give two lightweight Meas instances suitable for RAM that encrypt and authenticate the tree nodes with strictly bounded data complexity per key. Meas-v1 uses the PRINCE cipher and derives a fresh key for each encryption block using the sponge Ascon. Meas-v2 provides faster tree traversal by using the same key for the encryption of several, but a sufficiently small number of, e.g., 4 or 8, blocks using the tweakable cipher QARMA.

Finally, we implement both Meas instances on the Xilinx XC7Z020 System on Chip (SoC) Field Programmable Gate Array (FPGA) to practically evaluate the performance of RAM encryption and authentication with Meas.^1^ We show that Meas provides protection against the very powerful DPA attacks, and still features the same performance and memory overhead as state-of-the-art memory authentication schemes which completely lack side-channel protection. In particular, we show that a 4-ary, first-order DPA-secure instance of Meas-v2 is a highly suitable choice for encrypting and authenticating RAM in practice. Contrary to that, protecting cryptographic implementations against DPA to make use of state-of-the-art schemes would result in massive overheads making memory encryption and authentication infeasible.

### Outline

This work is organized as follows. In Sect. 2, we first state our threat model and requirements, and we then discuss the state of the art on memory encryption and authentication. The state of the art on side-channel attacks and countermeasures is content of Sect. 3. We analyze the re-keying countermeasure in terms of memory encryption in Sect. 4 and use the results to present our first-order DPA-secure Meas in Sect. 5. Section 6 then presents data masking to achieve higher-order DPA security in Meas. We give two lightweight instances of Meas suitable for RAM in Sect. 7 and detail their implementation in Sect. 8. An evaluation of Meas is done in Sect. 9, and we finally conclude in Sect. 10.

## Memory encryption and authentication

The encryption and authentication of memory is an important measure to prevent attackers with physical access from learning and/or modifying the memory content. There are several schemes for memory encryption and authentication available, but none of them takes the risk of side-channel attacks into account.

In this section, we define two threat models: the *non-leaking chip model* restates the state of the art [17, 25, 44, 48, 54, 55], and the extended *leaking chip model* further takes side-channel leakage into account. Moreover, we summarize present techniques for memory encryption and authentication and its requirements.

### Threat model and requirements

The *non-leaking chip model* in previous works assumes a single, secure microchip performing all relevant computations, e.g., a CPU. An attacker cannot perform any kind of active or passive attacks against this chip. All other device components outside this chip, e.g., buses, RAM modules and HDDs, are under full control of the adversary. Therefore, a physical attacker can, e.g., probe and tamper with buses, exchange peripherals, or turn the whole device on and off. For off-chip memory, this means that an attacker with physical access is capable of freely reading and modifying the memory content.

While reading can give an attacker access to confidential data stored inside the memory, modification breaks memory authenticity in several ways [17]: In *spoofing attacks*, an attacker simply replaces an existing memory block with arbitrary data, in *splicing attacks*, the data at address *A* is replaced with the data at address *B*, and in *replay attacks*, the data at a given address is replaced with an older version of the data at the same address.

Our *leaking chip model* extends the *non-leaking chip model* by considering passive side-channel attacks. It assumes that the microchip performing all relevant computations leaks information on the processed data via side channels, e.g., power and electromagnetic emanation (EM). Physical attackers can observe this leakage and perform side-channel attacks.

Hence, cryptographic schemes protecting the confidentiality and authenticity of off-chip memory in the *leaking chip model* have to fulfill three main requirements.

1. The only information an adversary can learn from memory is whether a memory block (i.e., ciphertext) has changed or not.
2. Prevention of spoofing, splicing, and replay attacks.
3. Protection against side-channel attacks.

In addition, fast random access to all memory blocks, high throughput (fast bulk encryption), and low memory overhead are desired.

### Memory encryption

Memory encryption schemes usually split the memory address space into blocks of predefined size, e.g., sector size, page size, or cache line size. Each of these blocks is then encrypted independently using a suitable encryption scheme. The partitioning of the address space into memory blocks aims to provide fast random access on block level and fast bulk encryption within the instantiated encryption scheme. Hereby, the chosen block size strongly affects possible trade-offs w.r.t. metadata overhead, access granularity, and speed.

So far, several memory encryption schemes have been proposed in the *non-leaking chip model* and are being used nowadays, e.g., the tweakable encryption modes XEX [47] and XTS [30], CBC with ESSIV [20], and counter mode encryption [48, 54].

### Memory authentication

Like for memory encryption, memory authentication schemes split the memory address space into blocks and aim for separate authentication of each of these blocks. Several memory authentication schemes have been proposed in the *non-leaking chip model*.

For example, a keyed Message Authentication Code (MAC) using the block address information can protect against spoofing and splicing attacks. However, it still allows for replay attacks. In order to protect against replay attacks, authenticity information must be stored in a trusted environment, e.g., in secure on-chip memory, that an attacker cannot modify. Authentication trees minimize this demand for secure on-chip storage, namely only the tree’s root is stored in secure memory, while the remaining tree nodes can be stored in public memory. Such trees therefore protect against spoofing, splicing, and replay attacks. Authentication trees over *m* memory blocks with arity *a* have logarithmic height $l=\log _a(m)$. Three prominent examples of authentication trees are Merkle trees [40], Parallelizable Authentication Trees [27] (PAT), and Tamper Evident Counter [18] (TEC) trees. We give a detailed description of them in Appendix A.

## Side-channel attacks

Present memory encryption and authentication schemes are designed to protect off-chip memory against adversaries with physical access assuming a microchip that is secure against all active and passive attacks. However, in IoT scenarios, the assumption that the microchip is secure against all passive attacks is often too strong since, in practice, a microchip running an algorithm leaks information on the processed data via various side channels, such as power, timing, and electromagnetic emanation (EM). This allows adversaries to perform passive side-channel attacks, which can reveal secret keys that are used in cryptographic implementations. There exist two basic classes of passive side-channel attacks [34]: Simple Power Analysis (SPA) and differential power analysis (DPA). Originally, SPA and DPA have been introduced for the power side-channel, but their basic principle is applicable to all kinds of side channels such as power, EM, and timing. Therefore, we will use the terms SPA and DPA throughout the paper, but note that our elaborations apply to all kinds of side channels.

### Simple power analysis

In SPA attacks, the adversary tries to learn the secret value processed inside a device from observing side channels during a single processing of the secret value to be revealed, e.g., the adversary tries to learn the encryption key from a power trace observed during a single encryption. However, the adversary is allowed to observe the same encryption multiple times to reduce measurement noise. Clearly, an implementation that cannot keep a key secret for a single encryption is worthless. Therefore, bounded side-channel leakage for a single encryption and thus security against SPA attacks is a precondition for any implementation.

### Differential power analysis

Quite naturally, the amount of information learned about a secret value from a side channel increases with the number of different inputs processed under the respective secret. This is exploited in DPA attacks, which use the observation of several different processings of a secret value in a device to learn its value, e.g., the adversary tries to learn the secret key from power traces observed during the encryption/decryption of multiple (public) input values.

One important property of DPA attacks is their order. The order *d* of a DPA [34, 41] is defined as the number of *d* different internal values in the executed algorithm that are used in the attack. The attack complexity of DPA grows exponentially with its order [12].

### Profiled attacks

Independently of whether SPA or DPA is performed, side-channel attacks can make use of profiling. Profiling of a side-channel, e.g., the power consumption, means to construct templates [13] that classify the side-channel information of a target device with respect to a certain value processed inside the device. In the actual attack, the templates are matched with the side-channel trace to gain some information on the value processed inside the device. The information learned from template matching can then be exploited in either SPA or DPA manner. Note, however, that conducting profiled attacks requires much more effort than performing non-profiled attacks. Further note that in many applications it is impossible to perform the required profiling step at all.

### DPA countermeasures

The effectiveness of DPA attacks has caused a lot of effort to be put into the development of countermeasures to prevent DPA. Two basic approaches to counteract DPA have evolved, namely (1) to protect the cryptographic implementation using mechanisms like masking, and (2) the frequent re-keying of unprotected cryptographic primitives.

#### Masking

Masking [12, 23], also called secret sharing, is a technique that can hinder DPA attacks up to certain orders. The idea behind masking is to prevent DPA by making the side-channel leakage independent from the processed data.

In a masked cryptographic implementation, every secret value *v* is split into $d+1$ shares $v_0,\ldots ,v_d$ in order to protect against *d*-th order DPA attacks. Thereby, *d* shares are chosen uniformly at random and the $(d+1)$-th share is chosen such that the combination of all $d+1$ shares gives the actual secret value *v*. As a result, an adversary is required to combine the side-channel leakage of all $d+1$ shares to be able to exploit the side channel, i.e., to perform a $(d+1)$-th order DPA.

While the masking operation itself is usually cheap, e.g., XOR, cryptographic primitives typically contain several operations that become more complex in the masked representation. This eventually results in massive implementation overheads. For example, the first-order DPA-secure threshold implementations of AES in [10, 42] add an area-time overhead of a factor of four.

#### Frequent re-keying

The success rate of key recovery with DPA rises with the number of different processed inputs. Therefore, frequent re-keying [33, 39] tries to limit the number of different processed inputs per key, i.e., the data complexity.

The countermeasure constrains a cryptographic scheme to use a certain key *k* only for *q* different public inputs (*q*-limiting [53]). When the limit of *q* different inputs is reached, another key $k'$ is chosen. Thus, for a certain key *k*, an adversary can only obtain side-channel leakage for *q* different inputs, which limits the feasibility of DPA to recover *k*.

Therefore, designing schemes and protocols with small data complexity *q* is one measure to prohibit DPA against unprotected cryptographic implementations. In more detail, it is widely accepted that very small data complexities, i.e., $q=1$ and $q=2$, have sufficiently small side-channel leakage and do not allow for successful key recovery from DPA attacks [7, 46, 53, 56].

*Leakage-resilient cryptography* Frequent re-keying can be applied to any cryptographic scheme, e.g., an encryption scheme *ENC* or an authenticated encryption scheme *AE*, by choosing a new key whenever a new message has to be encrypted and authenticated, respectively. However, in such a re-keying approach, side-channel resistance is also affected by the concrete instance of the cryptographic scheme. In practice, the cryptographic scheme must be able to process arbitrarily long messages using a standard primitive, e.g., AES with 128-bit block size. This situation facilitates DPA in certain modes, such as CBC. Therefore, the cryptographic scheme must be designed with special care.

**Fig. 1:**
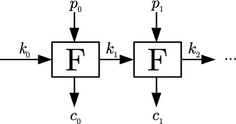
Generic encryption scheme *ENC*

A generic construction for an encryption scheme *ENC* that can process arbitrarily long messages without DPA vulnerability is given in Fig. 1. For DPA security, it requires a new key $k_0$ to be chosen for every new message. To securely process an arbitrary number of message blocks, the depicted scheme chains a primitive *F* that encapsulates the block encryption $c_i = E(k_i; p_i)$ and a key update mechanism $k_{i+1} = u(k_i)$. Hereby, the included key update mechanism $k_i \rightarrow k_{i+1}$ ensures the unique use of each key $k_i$. The construction can be considered secure against side-channel attacks if the key update mechanism is chosen such that the side-channel leakages of all invocations to *F* cannot be usefully combined. However, note that given that the key is iteratively derived using *F*, random access to individual blocks is typically quite expensive.

Exemplary constructions following the principle of Fig. 1 to design DPA-secure schemes from unprotected primitives are the leakage-resilient encryption schemes in [46, 53, 56] and the leakage-resilient MAC in [45]. Block-cipher-based instantiations of these schemes have a data complexity of $q=2$ in order to prohibit successful key recovery via DPA attacks.

## Re-keying for memory encryption

Frequent re-keying is a mechanism to protect against DPA without requiring that the implementation of the cryptographic primitive uses costly DPA countermeasures such as masking. Simultaneously, there are more and more practical systems being deployed with unprotected cryptographic accelerators by vendors not being aware of side-channel attacks. As a result, re-keying-based schemes are an interesting option for protecting memory encryption and authentication against DPA.

In this section, we perform the first investigation of the security of re-keying in the context of memory encryption and authentication. It shows that contrary to other use cases, the re-keying operation itself can be realized without DPA countermeasures when protecting memory. However, we also show that the application of re-keying to memory encryption allows for profiled, higher-order DPA that leaks confidential constants in memory due to read–modify–write operations inevitably occurring in encrypted memory.

### The re-keying operation

Up until now, the principle of re-keying was applied only to communicating parties aiming for confidential transmission. Hereby, constructions following Fig. 1 prevent DPA, but require the initialization with a fresh key and thus secure key synchronization between the communicating parties. A common approach to this synchronization is to derive a fresh key from a shared master secret *k* and a public, random nonce *n* [21, 39, 53]. However, this approach shifts the DPA problem to the key derivation, which thus needs DPA protection through mechanisms like masking.

The encryption and authentication of data stored in memory gives different conditions for the instantiation of re-keying-based schemes. In particular, encrypting data in memory means that encryption and decryption are performed by the same party, i.e., a single device encrypts data, writes it to the memory, and later reads and decrypts the data. Therefore, key synchronization becomes unnecessary and the cryptographic scheme can be re-keyed using random numbers without the need for any cryptographic primitive or function being implemented with DPA countermeasures.

### Re-keying and plaintext confidentiality

The typical target of DPA attacks is the key being used as key recovery fully breaks a cryptographic scheme. Re-keying-based schemes thus thwart such attacks and make DPA on the key infeasible. However, the actual goal of encryption is to ensure data confidentiality. Therefore, protecting the key against DPA is a useful measure, but as our analysis shows, the application of re-keying to memory encryption can yet result in a loss of memory confidentiality.

The main observation that leads to this conclusion is read–modify–write operations that inevitably occur in any encrypted memory. These take place whenever the write granularity is smaller than the encryption granularity. For example, when a single byte is written to a memory that is encrypted using an 128-bit block cipher, the respective 128-bit encryption block has to be loaded from memory, decrypted and modified according to the byte-wise write access, and then be encrypted again and written back to the memory. In this case, 120 bits of the respective block remain the same. The same phenomenon is observed in encryption schemes that cover multiple encryption blocks $p_0,p_1,p_2,\ldots$ . Here as well, one plaintext block, e.g., $p_0$, might be changing, while others, e.g., $p_1$, remain constant.

If now re-keying is applied to memory encryption, the constant plaintext parts within read–modify–write operations will be encrypted several times using different keys. This causes constant, secret plaintext parts to be mixed with varying keys. This situation is quite similar to the original DPA setting, where a constant, secret key is mixed with varying plaintexts. For stream ciphers, attackers can easily exploit this mixing operation—the XOR of varying pad and constant plaintexts—in a first-order DPA. Namely, attackers can model the power consumption of the varying pad for each plaintext hypothesis using the observed ciphertexts. Matching the power model with the side-channel observations eventually reveals the constant plaintext. For block ciphers, a first-order DPA does not work, but a profiled, second-order DPA that is similar to unknown plaintext template attacks [28] can be applied to learn constant plaintexts. While we emphasize that there are also other second-order techniques, e.g. [14, 35], that can be employed in this setting, we consecutively focus on adapting unknown plaintext template attacks to extract constant plaintexts from re-keyed block ciphers.

#### Unknown plaintext template attacks

In [28], the constant key *k* of a block cipher *E* is attacked by observing the encryption of several unknown plaintexts with the help of power templates. Hereby, the power templates are used to learn information on the unknown plaintexts $p_0,p_1,\ldots$ and intermediate values $v_0,v_1,\ldots$ in the respective encryption processes $E(k;p_0),E(k;p_1),\ldots$ . Exploiting the relation between the information learned on $p_0,p_1,\ldots$ and $v_0,v_1,\ldots$, the key *k* is recovered. As the attack combines side-channel information from both the unknown plaintexts $p_0,p_1,\ldots$ and the intermediate values $v_0,v_1,\ldots$, the order of this attack is two.

The described attack can be easily applied to a re-keyed encryption scheme (cf. Fig. 1). Namely, read–modify–write operations cause a constant plaintext block $p_i$ to be encrypted several times using different keys $k_i,k'_i,\ldots$ . Changing the roles of plaintext and key in the attack from [28], re-keying allows to learn the constant plaintext block $p_i$ from side-channel information on the varying key $k_i,k'_i,\ldots$ and some intermediate value $v_i,v'_i,\ldots$, both extracted using power templates. As a result, one plaintext may only be encrypted with one single key for re-keying to completely thwart DPA. This also seems reasonable in the view of leaking more information on a plaintext, the more often it is encrypted under different conditions, i.e., using different keys.

Summarizing, memory encryption inevitably causes read–modify–write operations. These cause re-keyed stream ciphers to become vulnerable to first-order DPA and re-keyed block ciphers to become vulnerable to profiled, second-order DPA. These attacks do not target the actual keys, but the confidential memory content. While these attacks cannot be prevented in the memory scenario, note that the effort and complexity of profiled, second-order DPA attacks is very high in practice. Hence, re-keyed block encryption provides a suitable basis to construct a memory encryption scheme with first-order DPA security. We further pursue this approach in Sect. 5. To obtain higher-order security, we extend our design in Sect. 6 and propose masking of the stored plaintext values. This effectively increases the number of values to be recovered via templates without the need for masking being implemented in the cipher.

## DPA-secure memory encryption and authentication

The analysis in Sect. 4 showed that frequent re-keying of a block-cipher-based mode is a suitable approach to construct a memory encryption and authentication scheme with first-order DPA security from unprotected cryptographic primitives. However, one major requirement in Sect. 2 is to provide fast random access in memory, but random access is not a feature of present re-keying-based encryption schemes.

A common way to provide fast random access to large memory is to split the memory into blocks that can be directly accessed. However, encrypting each of these memory blocks by the means of fresh re-keying would render the number of keys to be kept available in secure on-chip storage too high. This problem is quite similar to memory authentication with replay protection, which also requires block-wise authenticity information to be stored in a trusted manner. To tackle this issue, state-of-the-art authenticity techniques (cf. Sect. 2 and Appendix A) employ tree constructions to gain scalability and to minimize the required amount of expensive on-chip storage.

In this section, we therefore use the synergies between frequent re-keying and memory authentication to present Meas—a Memory Encryption and Authentication Scheme with first-order DPA security built upon unprotected cryptographic primitives and suitable for all kinds of large memory, e.g., RAM and NVM. Similar to existing memory authentication techniques, Meas uses a tree structure to minimize the amount of secure on-chip storage. However, instead of hashes or nonces, keys are encapsulated within the tree. In more detail, the leaf nodes of the tree, which store the actual data, are encrypted and authenticated using an authenticated encryption scheme that is provided with fresh keys on every write access. Similarly, the inner nodes of the tree, which store the encryption keys for their respective child nodes, are encrypted with an encryption scheme that uses a fresh key on every write. Meas is shown secure in the *leaking chip model*, and in particular, its DPA security is substantiated by limiting the number of different processed inputs per key to $q=2$ such as in [7, 46, 53, 56].

In the following, we first present the construction of Meas, followed by a security analysis considering authenticity and side-channel attacks.

### Construction

The construction of Meas is designed to be secure according to the *leaking chip model*. Therefore, Meas requires an SPA-secure block encryption scheme *ENC* and an SPA-secure authenticated encryption scheme *AE*. Both *ENC* and *AE* have to fulfill the common security properties for (authenticated) encryption schemes and must be based on block encryption such as in [56]. However, Sect. 7 will detail concrete instances for both *ENC* and *AE*. Apart from that, any other operations within Meas, such as loading keys, must be SPA-secure. In addition, a secure random number generator is needed for generating keys.

**Fig. 2:**
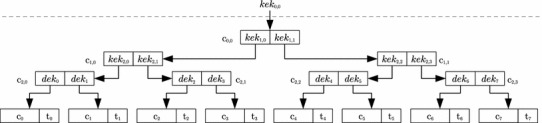
Meas ’s tree construction for $m = 8$ data blocks and with an arity of $a = 2$

An example of the tree construction proposed for Meas is depicted in Fig. 2. For the sake of simplicity, this example as well as the following description assumes the use of a binary tree, i.e., arity $a = 2$. However, instantiating the tree with higher arity is easily possible.

The structure of Meas is as follows. The data in memory is split into *m* plaintext blocks $p_i$. Each of these $p_i$ is encrypted and authenticated to a ciphertext-tag pair $(c_i, t_i)$ using the authenticated encryption scheme *AE* with data encryption key $dek_{i}$:

$$\begin{aligned} (c_{i}, t_{i})&= AE(dek_i; p_i)&\quad 0&\le i \le m-1. \end{aligned}$$

The encryption scheme *ENC* then encrypts the data encryption keys $dek_i$ to the ciphertexts $c_{l-1,i}$ using key encryption keys $kek_{l-1,i}$. The operator || denotes concatenation.

$$\begin{aligned} c_{l-1, i}&= ENC(kek_{l-1,i}; dek_{2i} || dek_{2i+1})&\,\,\, 0&\le i \le \frac{m}{2}-1. \end{aligned}$$

Recursively applying *ENC* in a similar way to the key encryption keys finally leads to the desired tree.

$$\begin{aligned} c_{j, i}&= ENC(kek_{j,i}; kek_{j+1,2i} || kek_{j+1, 2i+1}) \\&\quad 0 \le j \le l-2 , \quad 0 \le i \le \frac{m}{2^{l-j}}-1. \end{aligned}$$

While all ciphertexts and tags are stored in public, untrusted memory, the root key $kek_{0,0}$ is stored on the leaking chip.

#### Read operation

When reading data $(c_i, t_i)$ from memory, all the keys on the path from the root key $kek_{0,0}$ down to the respective data encryption key $dek_{i}$ are decrypted one after another. The data encryption key $dek_{i}$ is then used to decrypt and authenticate the respective memory block $(c_i, t_i)$.

For example in Fig. 2, to obtain the plaintext block $p_2$ stored in $(c_2,t_2)$, the root key $kek_{0,0}$ is used to decrypt $kek_{1,0}$. Then, $kek_{1,0}$ is used to decrypt $kek_{2,1}$, which permits to decrypt $dek_{2}$. Finally, $dek_{2}$ is used with $(c_2, t_2)$ to authenticate and decrypt the respective plaintext $p_2$.

Note that the decryption of the encapsulated keys can only be performed sequentially. However, this is not considered a problem since computation is typically much faster than storage (e.g., RAM or HDD). On the other hand, caching of the intermediate nodes (key encryption keys) is supported by Meas in order to achieve good performance, e.g., small average access latency.

#### Write operation

Writing data to the memory is where the actual re-keying is performed. Namely, the process of updating $p_i$ with $p_i'$ involves the replacement of all keys along the path from the root key $kek_{0,0}$ down to the respective data encryption key $dek_{i}$ with randomly generated ones. On the other hand, the keys for the adjacent subtrees are only reencrypted under the new node keys. This re-keying can be performed in a single pass from the root to the leaf node of the tree.

For example in Fig. 2, when block $p_5$, which is stored in $(c_5,t_5)$, gets replaced, also the keys $kek_{0,0}$, $kek_{1,1}$, $kek_{2,2}$ and $dek_5$ have to be changed. Therefore, the node $c_{0,0}$ is decrypted to extract $kek_{1,0}$ and $kek_{1,1}$. The new node $c_{0,0}'$ can then be determined by encrypting $kek_{1,0}$ and a new $kek_{1,1}'$ with the new key encryption key $kek_{0,0}'$. The nodes $c_{1,1}$ and $c_{2,2}$ are updated in the same way. The new data block $(c_5',t_5')$ is then the result of authenticated encryption of $p_5'$ under the new data encryption key $dek_5'$.

Note that it is not necessary to check authenticity when a full block is written to the memory. Only read–modify–write operations on a data block require an authenticity check. This authenticity check is automatically performed when the data is read prior to modification and thus does not inhere any additional costs. Also note that read–modify–write operations require only one single tree traversal, because the data encryption key required for the read operation automatically becomes available in the last steps of the write (and re-keying) procedure.

### Authenticity

The design of Meas protects data authenticity with respect to spoofing, splicing, and replay attacks using both the authentic root key and the *AE* scheme. In particular, spoofing and splicing attacks on the leaf nodes are directly detected by the *AE* scheme since different keys are used for each block. Moreover, the *AE* scheme indirectly also protects the inner tree nodes for properly chosen schemes *AE* and *ENC*. In such case, any tampering with the ciphertext of an intermediate node will lead to a random but wrong key to be decrypted. This tampering will thus propagate down to the leaf node to give an erroneous, random data encryption key and finally an authentication error.

Replay protection for all nodes is the result of the authentic root key, which gets updated on every write to any leaf node, i.e., choosing a new, random root key on every write access ensures that the secure root reflects the current state of the tree in public memory. Vice versa, the authenticity tags in the leaf nodes output by the *AE* scheme reflect the authenticity of the path from the root to the respective data block. Therefore, if the authenticity check of a leaf node fails, any node on the path from the root to the leaf may be corrupted.

#### Handling corruption

Our approach to verify the authenticity within Meas also has a strong influence on how data accesses need to be performed to be side-channel secure. In particular, Meas relies on two schemes *ENC* and *AE* that, for any tree node size, use each (internal) key only for a small number of, e.g., $q=2$, encryption blocks. To guarantee this property of *ENC* and *AE* also when accessing a certain leaf node within Meas, a secure implementation must only decrypt those plaintext parts within intermediate tree nodes (i.e., keys) that are actually needed for accessing the requested data block in the leaf. Namely, these keys become authenticated when accessing the leaf node, which allows to detect malicious modifications of these keys in memory. Eventually, this allows to identify attackers who perform DPA attacks on encryption keys by introducing authenticity failures on purpose. On the other hand, decrypting keys (in intermediate tree nodes) that are not used any further allows attackers to modify the respective keys’ ciphertext and thus to induce a DPA setting without being detected [15]. Nevertheless, when a corrupted leaf node has been detected, the authenticity of the tree must be restored before any further actions are taken.

Restoring authenticity of the tree is simple and requires no additional support. It is sufficient to replace all corrupted data (leaf) nodes with random values since regular writes restore authenticity from the root to the respective leaf node. Restoring authenticity in this manner also causes re-keying on all nodes on the path from the root to the leaf to take place. This re-keying procedure effectively thwarts any DPA that otherwise could be performed by malicious modification of stored ciphertexts.

For example in Fig. 2, if the authenticity check of the node $(c_4,t_4)$ fails, any of the nodes $c_{0,0}, c_{1,1}, c_{2,2}$ and $(c_4,t_4)$ can be erroneous. Therefore, the plaintext $p_4$ is replaced with a random plaintext $p'_4$ in order to restore the authenticity. Hereby, new keys $kek'_{0,0}, kek'_{1,1}, kek'_{2,2}$ and $dek'_4$ are chosen and the stored values $c'_{0,0}, c'_{1,1}, c'_{2,2}$ and $(c'_4,t'_4)$ are updated accordingly. This procedure restores the authenticity of the path from $kek_{0,0}$ to $dek_4$, but leaves any adjacent subtree intact. Moreover, the choice of fresh keys $kek'_{0,0}, kek'_{1,1}, kek'_{2,2}$ and $dek'_4$ prevents first-order DPA through adversaries repeatedly modifying $c'_{0,0}, c'_{1,1}, c'_{2,2}$ or $(c'_4,t'_4)$.

#### Recovering from corruption

Depending on the actual application, there are different approaches to deal with the corruption. A straightforward approach, which is suitable for RAM encryption, is to simply reset the tree and start from scratch. The memory encryption engine of SGX [25], for example, follows this approach and requires a system restart to recover. However, applying this idea to block-level disk encryption is impractical since a reset of the tree is equivalent to destroying the data of the whole block device.

Another, more graceful approach is to recover from the corruption when possible. In the case of RAM encryption, it is, for example, possible that the operating system kills (and restarts) only those processes which actually accessed a corrupted data block. In the setting of disk encryption, it can be enough to report which files or directories were destroyed to enable appropriate error handling.

Given a single authentication failure, it is not possible to determine which node is corrupted. However, since corruptions in higher tree nodes lead to authenticity failures in more data blocks, it is possible to identify the subtree which is affected by the data corruption using multiple adjacent reads. This can even be done quite efficiently in a binary search like approach (i.e., $\mathcal {O}(\log m)$ reads), assuming that only a single node has been corrupted.

For example in Fig. 2, when the authenticity check of data block 2, i.e., $(c_2,t_2)$, fails, then data block 3 is checked next. If block 3 is authentic, then only block 2 (child of $dek_2$) is corrupted. Otherwise, either block 0 or block 1 is checked next. If this next block is authentic, then only blocks 2 and 3 (children of $kek_{2,1}$) have been corrupted. In case of another error, a final check in the right subtree (children of $kek_{1,1}$) is needed to determine if only the left subtree (children of $kek_{1,0}$) or the whole tree is corrupted. Note, however, that locating the corruption requires each authenticity failure to be followed by a re-keying step as described in Sect. 5.2.1 in order to resist DPA. For example, if data block 2 is read and detected to be corrupted, the path from the root key to data block 2 must be re-keyed. If during the location phase data block 3 is detected to be unauthentic as well, also the path from the root key to data block 3 must be re-keyed. The same procedure applies to all other checks in the location phase.

### Side-channel discussion

We discuss the side-channel security using three types of attackers with increasing capabilities. The first type solely uses passive attacks and tries to exploit the side-channel leakage during operation. The second type additionally induces authenticity errors by tampering with the memory and strives for exploiting error handling behavior. The third type further tries to gain an advantage by restarting, i.e., power cycling, the whole system at arbitrary points in time.

#### Passive attacks

The protection of Meas against DPA lies within the re-keying approach. Therefore, every randomly generated key is used for the encryption and decryption of exactly one tree node with one specific plaintext. As soon as the plaintext of a node changes in any way, also a new key for the encryption of the respective node is generated.

For a certain key, a physical attacker who only passively observes Meas can thus at most acquire side-channel traces of one encryption and arbitrarily many decryptions of one single plaintext. Even though the trace number is possibly high, the best an attacker can do is to combine all the traces to a single rather noise free trace of this one key-plaintext pair. To perform a DPA, on the other hand, traces for multiple different plaintexts are required. In the presence of a passive physical attacker, Meas is therefore secure against first-order DPA attacks given that both *ENC* and *AE* are SPA secure.

#### Passive attacks and memory tampering

An active physical attacker who tampers with the memory content can gain additional information by corrupting the ciphertext of certain nodes. Namely, such tampering gives side-channel information from the decryption of different data for one single key. However, even with such tampering it is only possible to acquire one additional side-channel trace for a specific key. This is due to the fact that every tampering is detected as soon as the leaf node is authenticated. Handling the authentication error involves restoring authenticity and thus re-keying which makes the gathering of further traces impossible. As a result, the number of acquirable traces (i.e., under the same key, but with different ciphertexts) is clearly bounded by two. Given the assumptions in related work on leakage-resilient cryptography [46, 53, 56], bounding the input data complexity per key by two makes Meas secure against first-order DPA for malicious memory corruption.

#### Passive attacks, memory tampering and restarts

The side-channel security of Meas relies on the assumption that tree operations are performed atomically. This means that, e.g., once a read operation is started, all steps involved in Meas, i.a., the MAC verification and the re-keying on authenticity failure, must be performed and completed. This assumption holds true for a running device since physical fault attacks on the leaking chip are outside the threat model. However, restarting the device during operation can break this assumption. In this case, attackers can use a combination of power cycling and memory tampering to collect arbitrarily many side-channel traces and perform a first-order DPA against a non-volatile key. However, this attack is easily prevented when the concrete use case is known.

For the encryption and authentication of RAM, there is simply no reason to maintain persistent keys between system restarts. Similar to SGX, the device generates a new random key on startup which effectively thwarts the attack. For NVM, however, a persistent root key is unavoidable. Yet, there are easy and secure solutions for NVM too. For example, one could store one additional bit on the leaking chip to record whether a presumably atomic operation is currently active. This allows to detect aborted operations in Meas on startup and thus to take further actions, e.g., counting and storing the number of aborted operations on the leaking chip and appropriate error handling when a certain threshold is reached. Such countermeasures can also be integrated with the transaction/journaling functionality of a file system.

Summarizing, Meas itself does not contain any mechanism to deal with malicious power cycling. However, for both RAM and NVM simple and cheap solutions are available.

## Higher-order DPA security

The tree construction presented in the previous section provides memory confidentiality and authenticity in the presence of a first-order side-channel adversary. However, profiled, second-order attacks as outlined in Sect. 4 still reveal the content of the tree nodes protected by the means of re-keying. Since the loss of confidentiality of a node close to the root would also reveal large chunks of the protected memory, i.e., all child nodes, protection against higher-order DPA is desirable.

In this section, we propose masking of the plaintext values to extend the protection of Meas to higher-order DPA. The extension works with cryptographic primitives implemented without DPA countermeasures and allows to dynamically adjust the protection order depending on the actual threat.

### Concept

The basic idea to provide higher-order DPA security is to add a masking scheme (cf. Sect. 3.4.1) to Meas. However, unlike the masking of specific cryptographic implementations, the proposed data masking scheme operates with unprotected primitives. Therefore, the plaintext data in each tree node of Meas is first masked, and then the masked plaintext and the masks are encrypted separately and both stored in memory. On decryption, both the masked plaintexts and the masks are decrypted and the masks applied to obtain the original plaintext value.

The masking scheme requires new masks to be chosen whenever the key of a tree node is changed. This is the case on every write access to a specific node. As a result, the data being encrypted is randomized. This prevents that constant data is encrypted under different keys. Moreover, it requires adversaries trying to learn a constant plaintext using profiled attacks such as described in Sect. 4 to additionally extract information on every single mask from the side-channel. Therefore, the order of the attack increases accordingly.

### Masking details

The following masking approach can be applied accordingly to both the intermediate nodes, which use an encryption scheme *ENC*, and the leaf nodes, which use an authenticated encryption scheme *AE*. However, for simplicity we only consider the encryption of an arbitrary tree node using an encryption scheme *ENC*.

When encrypting a tree node in Meas, the node’s plaintext *p* is split into $b+1$ blocks $p_0,\ldots ,p_b$ according to the size of the underlying encryption primitive, i.e., 128 bits in case of AES. In order to protect this node against *d*-th order DPA, $d-1$ random and secret masks $m_0,\ldots ,m_{d-2}$ have to be generated. These masks are then applied to each plaintext block $p_i$ to give random values $r_i$:

$$\begin{aligned} r_i&= p_i \oplus m_0 \oplus \cdots \oplus m_{d-2}&\quad 0&\le i \le b. \end{aligned}$$

In the actual encryption, both the masks $m_0,\ldots ,m_{d-2}$ and the random values $r_0,\ldots ,r_b$ are processed and the respective ciphertext *c* is stored in memory:

$$\begin{aligned} c = ENC(dek; m_0||\cdots ||m_{d-2}||r_0||\cdots ||r_b). \end{aligned}$$

Whenever the node has to be read, the ciphertext is decrypted to give $m_0||\ldots ||m_{d-2}||r_0||\ldots ||r_b$. To obtain the plaintext blocks $p_i$, the masking is reverted by again xor-ing all masks $m_0,\ldots ,m_{d-2}$ to each block $r_i$.

### Side-channel discussion

The re-keying of the (authenticated) encryption scheme guarantees that adversaries are not capable of building suitable DPA power models from the observation of ciphertexts and thus prevents DPA against the key completely.

To prevent the loss of plaintext confidentiality from the profiled, second-order attacks outlined in Sect. 4, the proposed masking scheme randomizes the plaintext input using $d-1$ random, secret masks. As a result, the scheme requires adversaries to combine side-channel information from $(d+1)$ different values to recover the plaintext, i.e., to perform a $(d+1)$-th order DPA. In particular, such DPA requires to learn side-channel information on the varying key, an intermediate value in the cipher, and the $d-1$ masks. On the other hand, the masking scheme requires to additionally encrypt $d-1$ masks in each tree node. However, for a properly chosen encryption scheme *ENC*, these encryption operations cannot be exploited in a DPA, because both the masks and the keys are random and always changed simultaneously on every write access to the respective tree node.

Unfortunately, using the same masks for multiple encryption blocks within a tree node can yet give side-channel leakages with an order below $d+1$. For illustration, we consider a single mask $m_0$, i.e., $d=2$. In this case, attackers could exploit the combination of $m_0$ with $b+1$ different plaintext blocks within a tree node to learn the mask $m_0$ in a second-order side-channel attack with unknown input and output. Once $m_0$ is known, $m_0$ can be used to learn a constant plaintext block $p_i$ in the same tree node using another second-order attack.

However, such lower-order attacks are impractical for Meas for two main reasons. First, in order to perform a lower-order attack on a constant $p_i$, an attacker must initially learn all the masks $m_0,m'_0,m''_0,\ldots$ as they are changed upon re-keying. Second, the number of different operations with a certain mask $m_0$ is bounded by the number of $b+1$ plaintext blocks in each tree node. For example, a 4-ary instance of Meas might reuse the same mask four times, which will typically not suffice to recover the mask. Consequently, the data complexity for each mask is limited in the same way as it is limited for each key within Meas, making lower-order attacks to learn the masks practically infeasible. Contrary to that, the number of different keys used for a constant plaintext block $p_i$ is potentially unlimited.

The data complexity for each mask depends on the number of plaintext blocks in a tree node that share the respective mask. This number of plaintext blocks depends on the tree arity *a* for intermediate nodes, and on the data block size $s_b$ for data leaf nodes. Hence, both the data block size and the tree arity must be chosen to give a data complexity per mask that suits the device’s leakage behavior. Hereby, note that learning the masks involves at least a second-order attack setting with unknown input and output, which usually allows for higher data complexities than for keys that encrypt/decrypt known plaintexts or ciphertexts.

From an implementation perspective, the sum of plaintext and the masks must be stored in a register prior to the encryption operation for the masking to protect Meas also in the presence of hardware glitches. This is automatically the case if the masking is implemented in software. Hereby, the result is stored in a register and may then, e.g., be further processed in a cryptographic hardware accelerator.

Besides, we also emphasize that profiled DPA attacks such as in Sect. 4—which are counteracted by the proposed masking scheme—are quite hard to conduct on state-of-the-art systems. For example, while the unknown plaintext template attack in [28] was performed against software implementations on 8-bit and 32-bit microcontrollers, a profiled DPA will take significantly more effort on hardware implementations embedded in a complex system-on-chip. Moreover, the attack complexity also rises rapidly with the attack order. As a result, small protection orders will already be sufficient for Meas in practice. However, a detailed analysis of the side-channel leakage of a device implementing Meas is indispensable for a proper choice of the protection order.

**Fig. 3:**
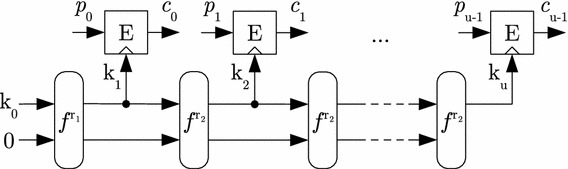
Schematic overview of ENC in Meas-v1

### Implementation aspects

The definite choice of the implemented protection order allows for various trade-offs influenced by several parameters: the cost for storing the masks, the concrete leakage behavior of the device, and the risk. Hereby, the leakage behavior and the cost for storing the masks are closely coupled.

A DPA is more likely to be successful on a device the more side-channel leakage the device gives. Therefore, a higher protection order is needed the more the device leaks, which leads to higher storage costs for masks. Alternatively, the leakage of the device might be reduced by hiding countermeasures [38] in the implementation, such as shuffling. However, such countermeasures can only be built into newly designed devices. Nevertheless, besides the actual strength of a potential attacker, the actual leakage behavior of the device forms the basis for the choice of the protection order and thus memory cost.


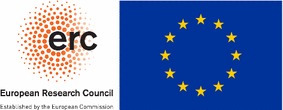


Besides, the choice of the protection order is also strongly influenced by the concrete risk of an attack. In more detail, a trade-off between the protection order and the risk is possible. Namely, the higher the risk of an attack to a specific block, the better should be the protection of the respective block, i.e., the higher should be the protection order. Concretely in Meas, the tree nodes stored in levels closer to the root are a more interesting target for an attacker since revealing the keys stored in these nodes would allow to decrypt large parts of the memory. Therefore, tree nodes closer to the root are at higher risk and thus need a higher protection order. However, the number of nodes in one tree level decreases the closer the respective level is to the root. As a result, increasing the protection order for tree nodes at higher risk has only little memory overhead in Meas and thus is an inexpensive improvement of security against higher-order DPA.

**Fig. 4:**
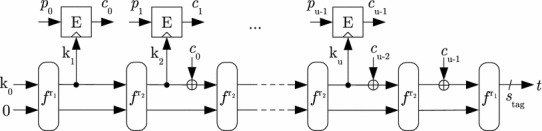
Schematic overview of *AE* in Meas-v1

## Instantiation

The design of Meas requires an SPA-secure block encryption scheme *ENC* and an SPA-secure authenticated encryption scheme *AE*. Using existing proposals of leakage-resilient block encryption [56] and a leakage-resilient MAC [45], both *ENC* and *AE* can be easily obtained from unprotected cryptographic implementations of standard primitives like AES and SHA-2 and the generic composition encrypt-then-MAC [8]. However, for the encryption and authentication of RAM, more lightweight constructions for *ENC* and *AE* are desirable.

In this section, we present two lightweight Meas instances for the purpose of RAM encryption and authentication. The first, Meas-v1, uses the lightweight block cipher PRINCE for encryption, and the sponge Ascon for key stream generation and authentication. As a result, Meas-v1 uses a fresh key for the encryption of each plaintext block to prevent DPA on the key. The second, Meas-v2, improves on Meas-v1 in terms of efficiency in trade for an slightly increased number of, e.g., 4 or 8, different inputs processed under the same key. In particular, it omits key derivation in intermediate tree nodes to instead directly access the required keys using the tweakable block cipher QARMA. The security of Meas-v2 thus relies on the infeasibility of DPA for 4- or 8-limited data complexity per key.

### Meas-v1

Our instance Meas-v1 is intended for RAM encryption and authentication and constructs *ENC* and *AE* by combining two different primitives: a lightweight block cipher *E* for encryption, and an *r*-round permutation $f^r$ for sponge-based key derivation and authentication. While *ENC* uses the sponge merely for key stream generation, the sponge duplex construction [9] is used in *AE* to also absorb the computed ciphertext and to compute the tag. Algorithm 1 gives the description of the respective algorithms. Their schematic is illustrated in Figs. 3 and 4, respectively. Since Meas applies (authenticated) encryption to message blocks of fixed, well-defined length, we describe *ENC* and *AE* without a padding rule and assume the messages to be a multiple of the *b*-bit block size of *E*. Note that for optimization, *AE* absorbs the ciphertexts $c_i$ with some delay. This allows to compute the permutation $f^{r_2}$ and the encryption *E* in parallel.


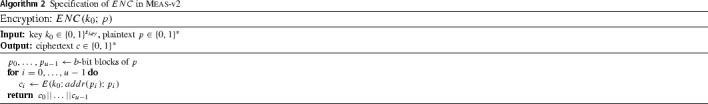


We use PRINCE [11] as the block cipher *E* and the Ascon permutation [16] with $r_1=8$ and $r_2=6$ rounds for the sponge. PRINCE uses a key of $s_{key}=128$ bits to process blocks of $b=64$ bits and the Ascon state *S* is sized $s_{state}=320$ bits. These parameters allow to implement both *ENC* and *AE* with adequate throughput and low latency in hardware. The size of the tag $s_{tag}$ can be chosen according to the desired security level, e.g., $s_{tag}=64$ or 128 bits.

### Meas-v2

One performance bottleneck of Meas-v1 is the sequential key derivation within a tree node. On the other hand, direct access to a certain key within an intermediate tree node can significantly increase performance. By relaxing the constraints for DPA security, direct access to certain keys within a specific tree node becomes feasible.

For this purpose, we construct *ENC* using a tweakable block cipher. This allows to efficiently encrypt/decrypt parts of a tree node similar to ECB, but provides better security in terms of ciphertext distinguishability. Given a tweakable block cipher $E(k;\tau ;p)$ that encrypts a *b*-bit plaintext *p* with the key *k* and tweak $\tau$, a tree node comprising *u* plaintext blocks $p_0,\ldots ,p_{u-1}$ is thus encrypted by simply computing $E(k; addr(p_i); p_i)$ for $i=0,\ldots ,u-1$, where the tweak $\tau$ is set to be the address of the respective $p_i$ in memory. This is summarized in Algorithm 2.

On the other hand, we keep the design of *AE* in Meas-v2 the same as in Meas-v1. However, to avoid the implementation of another cipher for the use in *AE*, we recommend using the same tweakable cipher $E(k; \tau ; p)$ in *AE* as well and set the tweak $\tau$ in *AE* to either the block address or a constant. As the tweakable block cipher $E(k; \tau ; p)$, we use the lightweight design QARMA-64 [4] with the parameter $r=6$.

In terms of DPA, the mentioned approach increases the number of different inputs processed using a single key according to the number of plaintext blocks *u* in a tree node. However, for many practical implementations DPA will remain infeasible also for, e.g., 4 or 8, different encryptions using the same key. This assumption facilitates the efficient and secure implementation of Meas-v2 for, e.g., binary and 4-ary trees.

## Implementation

The two lightweight instances Meas-v1 and Meas-v2 are designed for RAM encryption and authentication. In order to show their practical applicability to this use case, an implementation allowing the evaluation of performance and implementation cost is desirable. In this section, we thus present an implementation of both Meas instances on the Xilinx Zynq platform.

### Platform

For the implementation, we chose a ZedBoard featuring the Xilinx Zynq XC7Z020 SoC and 512 MB DDR3 RAM. This SoC consists of two parts: (1) a processing system (PS) comprising a dual-core ARM Cortex-A9 processor as well as several peripherals, and (2) a Xilinx Artix-7 programmable logic (PL). The PS is connected to the PL via 32-bit advanced extensible interfaces (AXI). The PL has access to the RAM via 64-bit AXI.

**Fig. 5:**
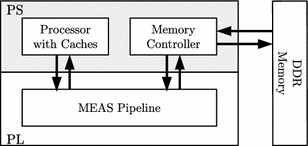
Zynq platform with Meas pipeline

For memory encryption and authentication, we designed an encryption pipeline capable of Meas that is placed in the PL. As shown in Fig. 5, the software running on the ARM core is configured such that the processor accesses the main memory via the PL, where all accesses are transparently encrypted and authenticated using Meas.

**Fig. 6:**
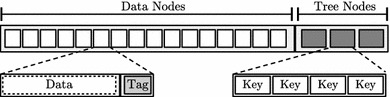
Memory layout for 4-ary Meas

**Fig. 7:**
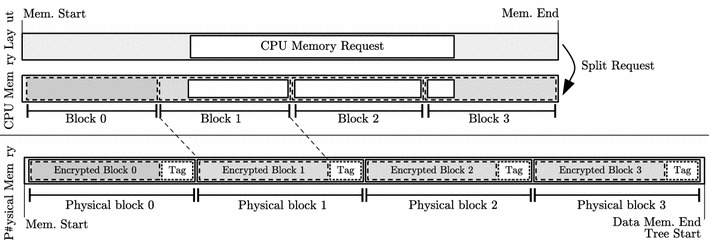
Data node requests for 4-ary Meas

### Memory layout

The implementation of Meas requires to place all the tree nodes as well as their metadata somewhere in the RAM. For this purpose, and as shown in Fig. 6, the physical memory is partitioned into two parts. In the first part, all data (leaf) nodes of Meas are placed. These also contain their respective authenticity tags. The consecutive, second part contains all intermediate tree nodes storing the keys.

### Address translation

In order to provide the functionality of Meas transparently to the CPU, a translation of the CPU’s memory requests to the encrypted physical memory is required. Without consideration of tree node fetches, Fig. 7 illustrates this CPU address translation. The CPU memory request is split according to the block size of the data (leaf) nodes. The Meas implementation then issues independent requests to each of these data leaf nodes. Hereby, the size of the authentication tags is taken into account, which causes both an address shift and additional tags to be fetched.

However, the tree construction requires to also load several keys to decrypt a certain data (leaf) node. These key load operations are handled the same way as the requests to the data leaf nodes themselves. In particular, the Meas implementation issues, translates, and processes the respective key load requests to intermediate tree nodes transparently without further CPU interaction and follows an address translation similar to data leaf nodes.

**Fig. 8:**
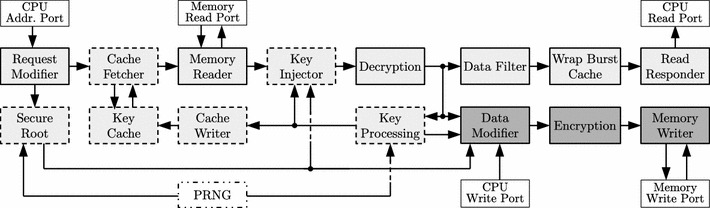
Meas encryption and authentication pipeline

### MEAS pipeline

The pipeline architecture of our Meas implementation is visualized in Fig. 8. Its design results from the typical data flow in encrypted memory accesses. In particular, all requests run through a series of modules performing different actions. Hereby, the single modules interact by using a simple handshake mechanism. The width *w* of the respective data stream can be set to either 64 or 128 bits.

Our implementation communicates with CPU and memory via five different AXI4 interfaces: (1) the CPU address port, (2) the CPU read port, (3) the CPU write port, (4) the memory read port, and (5) the memory write port. Read requests use the modules shaded in light gray. Write requests are implemented as read–modify–write operations and additionally use the modules depicted in dark gray. Dashed lines mark modules needed to process or optimize key-related requests to intermediate tree nodes.

#### Data flow

The implementation in Fig. 8 processes a typical memory request as follows. First, the CPU issues a request on the CPU Address Port. The Request Modifier then splits and aligns the request according to the block size of the data (leaf) nodes. It further issues the respective key load requests within intermediate tree nodes. The Memory Reader fetches the required (encrypted) data from the main memory via the Memory Read Port. The Key Injector then inserts the key to be used for decryption into the data stream fetched from memory. This key might either be a root key stored in the Secure Root, or be the result of a previous key load request that is obtained by the Key Processing module. The Decryption module performs the actual decryption procedure according to our instances in Sect. 7.

For key load requests, the requested key is extracted from the decrypted data using the Key Processing Module. For read requests, the decrypted data is filtered according to the original CPU request by the Data Filter and returned to the CPU via the CPU Read Port by the Read Responder. To correctly handle CPU read requests with wrapping burst functionality, the Wrap Burst Cache performs a re-ordering of the decrypted data if necessary. For write requests, the Data Modifier modifies the decrypted data according to the data received from the CPU via the CPU Write Port. This is where the actual read–modify–write procedure takes place. The modified data is encrypted again using the Encryption module and written to the main memory via the Memory Write Port by the Memory Writer.

To improve the performance of the Meas pipeline, the Secure Root can implement an arbitrary number of roots to support multiple parallel trees in memory. Multiple roots effectively reduce both the tree height and the memory overhead in case more secure memory is available on the secure chip. To further improve the performance of read requests, the Meas pipeline incorporates a Key Cache for faster key retrieval within the tree. For this purpose, the Cache Fetcher queries the cache for the key requested in a key load request. On a hit, the key load request is dropped and the key forwarded. Otherwise, the key load request is forwarded without modification. The Key Cache is filled using the Cache Writer, which receives the keys to be stored in the cache from the Key Processing module.

#### Re-keying

Write requests in Meas require the re-keying of all nodes on the path from the root to the respective data leaf node. This re-keying operation takes place in the Secure Root for the root keys themselves, and in the Key Processing module for non-root keys stored within the tree. In particular, besides filtering out the decryption keys from the decrypted data in key load requests, the Key Processing updates the respective keys during write requests. The new key is generated by the pseudo-random number generator PRNG. This PRNG uses a Keccak[400] instance that is initialized with a random secret and that securely sequeezes a secret, pseudo-random stream. The freshly generated keys are provided to the Data Modifier to update the keys in the respective write requests and for encryption.

**Table 1:** Comparison of Meas with other constructions for scalable authentic and/or confidential memory which offer block-wise random access

	Auth.	Conf.	DPA security	Parallelizable		Memory overhead
				Read	Write	
Meas	$\checkmark$	$\checkmark$	$\checkmark$			$\frac{a}{a-1} \cdot \frac{s_{key}}{s_b} + \frac{s_{tag}}{s_b}$
PAT	$\checkmark$			$\checkmark$	$\checkmark$	$\frac{a}{a-1} \cdot \frac{s_{tag}+s_{nonce}}{s_b}$
TEC tree	$\checkmark$	$\checkmark$		$\checkmark$	$\checkmark$	$\frac{a}{a-1} \cdot \frac{s_{tag}+s_{nonce}}{s_b}$
Merkle tree	$\checkmark$		$\checkmark$	$\checkmark$		$\frac{a}{a-1} \cdot \frac{s_{hash}}{s_b}$

## Evaluation

Meas is a novel approach to provide authentic and confidential memory with DPA protection. While there already exist several concepts for memory encryption and authentication (cf. Sect. 2), all of them lack the consideration of side-channel attacks.

In this section, we compare Meas with these state-of-the-art techniques regarding security properties, parallelizability, randomness, and memory overhead. Our methodology to assess the memory overheads is independent of any concrete implementation, precisely states the asymptotic memory requirements of all schemes, and is realistic for any real-world instance. In addition, we evaluate the practical performance of our Meas implementation from Sect. 8 compared to TEC trees when encrypting RAM. It shows that Meas efficiently provides first-order DPA-secure memory encryption and authentication at roughly the same memory overhead and performance as existing authentication techniques, which, on the other hand, completely lack the consideration of DPA at all. In particular, the 4-ary instance of Meas-v2 is a highly suitable choice for DPA-secure encryption and authentication of RAM.

### Security properties

Comparing the contestants in Table 1 regarding security properties shows that only Meas and TEC trees provide both confidentiality and authenticity in the form of spoofing, splicing and replay protection. DPA security, on the other hand, is only featured by Meas and Merkle trees. However, Merkle trees do not provide confidentiality and their DPA security can be considered a side effect. Namely, the hash functions used in Merkle trees simply do not use any secret material, i.e., keys or plaintexts, which is the common target in DPA attacks.

### Parallelizability

A more performance oriented feature, on which previous tree constructions typically improved on, is the ability to compute the cryptographic operations involved in read and write operations in parallel. Having this property is nice in theory, but is in practice not the deciding factor to gain performance. To make use of a scheme’s parallelism, multiple parallel implementations of the cryptographic primitives as well as multi-port memory, to read and write various nodes in parallel, are required. Since these resources are typically not available, a common, alternative approach to improve performance is the excessive use of caches.

In Meas, due to the key encapsulation approach used to achieve its DPA security, parallelizing the computations within the encryption scheme is not possible. However, this is not necessarily a problem preventing the adoption of Meas in practice since on-chip computation is very fast compared to off-chip memory accesses. Additionally, like for all authentication trees, caches for intermediate nodes are a very effective and important measure to reduce the average latency. In summary, the performance of any authentication tree (and Meas) is mainly determined by the tree height, which depends on both the tree arity and the number of blocks in the authenticated memory, and the cache size. As a result, given a concrete implementation of the cryptographic primitive, the actual runtime performance of all authentication trees is expected to be quite similar, which is also emphasized by the implementation results following in Sect. 9.6.

### Memory overhead

Table 1 further contains the memory overhead formulas that have been derived for each scheme. These formulas take into account the tree arity *a*, and the sizes for data blocks $s_b$, nonces $s_{nonce}$, hashes $s_{hash}$, tags $s_{tag}$, and keys $s_{key}$. The overhead formulas neglect the influence of the actual number of data blocks *m* given that it vanishes with rising node counts. The overheads therefore have to be considered as an upper bound which gets tight with $m \rightarrow \infty$. This approach gives exact and comparable results that are independent of the actual implementation and that are realistic for any memory with more than 128 data blocks.

The different parameters involved may make the overhead comparison seem difficult at first glance. However, it gets quite simple when actual instantiations are considered. Instantiating the trees for a fixed security level with $s_{nonce} = s_{tag} = s_{key}$ and $s_{hash} = 2 \cdot s_{tag}$, for example, shows that Merkle trees, PATs, and TEC trees have identical overhead. The overhead of Meas, on the other hand, is even lower, especially with small arity. This is due to the fact that in Meas only leaf nodes are directly authenticated. On the other hand, PATs and TEC trees directly protect the authenticity of every tree node.

**Fig. 9:**
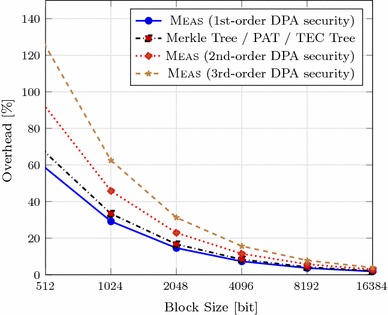
Memory overhead comparison for 4-ary trees depending on protection order and block size with a security level of 128 bits ($a=4$, $s_{nonce} = s_{tag} = s_{key} = 128$, $s_{hash} = 256$)

The memory overhead of Meas, PATs, Merkle trees, and TEC trees is also visualized in Fig. 9 for different block sizes. For practical instantiations, the block size will be chosen according to the system architecture, namely page size, sector size, or cache line size. Both the sectors of modern disks and memory pages in state-of-the-art systems are sized 4096 bytes $(=32768$ bits). Such large block size is out of scope of Fig. 9 as it has negligible memory overhead in any case. Besides, the memory overhead for a block size of 4096 bits (sector size in older hard disks) is also very low, e.g., 7.3% for 4-ary Meas. However, the memory overhead of Meas for block sizes fitting nowadays cache architectures is also practical given the security features it provides. While today’s typical cache line size is 512 bits, modern CPUs often come with features such as Adjacent Cache Line Prefetch [31], which effectively double the cache line fetches from memory to 1024 bits. In a 4-ary Meas, for example, such block size results in decent 29.2% memory overhead.

Note that these relatively small overheads—quite similar to existing authentication techniques—in combination with additional and exclusive DPA protection are the main advantage of Meas. Using existing memory encryption and authentication schemes with DPA-protected implementations, on the other hand, would result in overheads of a factor of four to a few hundred [6, 10, 42, 45] and thus be far more expensive, eventually rendering memory encryption and authentication in many applications impractical.

### Memory overhead with masking

The memory overhead of Meas with higher-order DPA protection additionally depends on the protection order *d* and the size of the masks $s_{mask}$. This size $s_{mask}$ typically equals the block size of the cryptographic primitive $s_{state}$. A generalized version of the limit of the memory overhead as the number of memory blocks approaches infinity is:

$$\begin{aligned} \frac{a}{a-1} \cdot \frac{s_{key} + (d-1)\cdot s_{mask}}{s_b} + \frac{s_{tag}}{s_b}. \end{aligned}$$

In addition to the memory overhead without masking, Fig. 9 shows the memory overhead with masking for a 4-ary tree and 128-bit security, i.e., the keys, the tags, and the masks are sized 128 bits. It shows that masking adds multiplicatively to the memory overhead for all block sizes. However, for larger block sizes, the memory overhead of Meas becomes negligible regardless of the protection order. Note that the protection order stated for Meas in Fig. 9 applies to all nodes in Meas. If, however, and as explained in Sect. 6.4, different protection orders are used for nodes at different risk, the depicted plots mark the border cases for the actual memory overhead. For example, if low-level tree nodes do not use masking (i.e., having first-order DPA security) and first-order masking is applied to all other nodes (i.e., having second-order DPA security), the actual memory overhead is lower- and upper-bounded by the plot with first- and second-order protection, respectively.

**Fig. 10:**
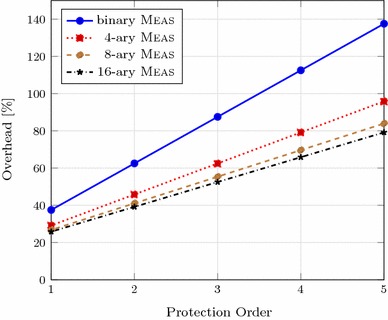
Memory overhead of Meas depending on arity and protection order (1024-bit blocks, 128-bit security)

An evaluation of the memory overhead of Meas over different protection orders and arity is depicted for 1024-bit blocks and 128-bit security in Fig. 10. Hereby, it turns out that the memory overhead is strongly influenced by the tree’s arity leading to two main observations. First, a higher arity clearly lowers the memory overhead, but for an arity higher than eight, the reduction resulting from another increase of the arity becomes quite small. Second, the memory overhead rises linearly with the protection order, but the increase is stronger the lower the tree’s arity is. This is due to the masks for randomization of the plaintext being chosen and stored for each tree node. As a result, higher arity leads to more plaintext blocks sharing such masks in one tree node and thus lower memory overhead due to the masking.

### Randomness

Meas consumes a considerable amount of randomness. In particular, fresh random keys and masks must be chosen for all nodes on the path from the root to the leaf whenever a write operation is performed. For Meas with protection order *d*, this sums up to $(s_{key} + (d-1)\cdot s_{mask}) \cdot (l+1)$ random bits needed on each write operation, where *l* is the tree height. Implementations of Merkle trees, PATs and TEC trees without consideration of side channels, however, do not require any random value if all nonces are chosen as counters. Yet, cipher implementations that protect PATs and TEC trees against side-channel attacks rely on significant amounts of randomness too. Namely, implementations with protection order *d* split their state into $(d+1)$ shares. This demands for at least $d \cdot s_{state}$ random bits per cipher invocation that get necessary for all accessed nodes on both reads and writes. Contrary to that, Meas does not require randomness during read accesses.

### Implementation results

We extensively evaluated the performance of our Meas implementation from Sect. 8. In particular, we ran both Meas-v1 and Meas-v2 on the Digilent ZedBoard using different tree arities. As a state-of-the-art reference, we further implemented and ran a variant of TEC trees with different arities based on the same architecture as given in Fig. 8. These TEC trees use Ascon  [16] for authenticated encryption. For all our evaluations, we used an unprotected implementation of Ascon that computes three permutation rounds per cycle.

The evaluations for TEC trees were done with 64-bit counters (nonces) and 64-bit tags, which is a common instance for TEC trees in RAM [25]. For our evaluations of Meas, we used a side-channel protection order $d=1$ and 128-bit keys. Besides, we operated Meas with 128-bit tags as 64-bit tags only gave negligibly better results. Another relevant evaluation parameter is the data block size $s_b$. A suitable choice for $s_b$ typically is the processor’s cache line size. While the cache of the ARM Cortex-A9 processor on the ZedBoard’s Xilinx XC7Z020 SoC features 256-bit cache lines, we configured the cache to always fetch 512-bits from memory by enabling the double line fill feature [3]. For this reason, both Meas and the TEC tree use a data block size of $s_b=512$ bits. To speed up our designs, we made use of 1024 root keys (or root nonces for TEC trees) and a cache with 1024 slots to store keys (or nonces, respectively).

All our implementations use the 32-bit GP0 AXI interface to the CPU and the 64-bit HP0 AXI interface to the memory. As a result, a natural choice for the width *w* of the internal data stream that connects the various modules in Fig. 8 is 64 bits. For the TEC tree implementation, we hence set $w=64$ bits. On the other hand, Meas operates heavily on 128-bit keys, which could make a 128-bit internal stream more efficient. For this reason, we evaluated the performance impact of the internal data stream width by running both Meas-v1 and Meas-v2 with both $w=64$ and $w=128$ bit internal stream width.

**Fig. 11:**
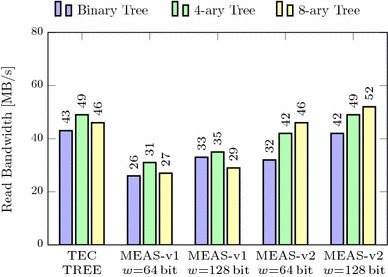
Read performance determined with tinymembench (NEON read prefetched (64 bytes step))

**Fig. 12:**
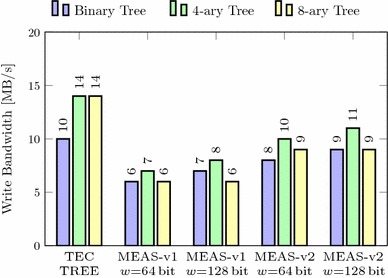
Write performance determined with tinymembench (NEON fill)

In our evaluations, we booted Linux (Xilinx Linux kernel 4.4, tag 2016.2) [59] in encrypted and authenticated memory, and measured the memory performance using a set of benchmarks. In particular, we executed tinymembench 0.3 [51] and LMBENCH 3.0-a9 [52] for determining the memory bandwidth and latency, respectively. We performed these benchmarks for 256 MB of encrypted and authenticated memory provided to the ARM CPU and an FPGA clock frequency of 50 MHz. Note that at 50 Mhz, the 32-bit GP0 interface bounds the achievable memory bandwidth with 200 MB/s.

#### Memory bandwidth

Figures 11 and 12 show the read and the write memory bandwidth for all our designs and different tree arities. As mentioned before, we compare both Meas-v1 and Meas-v2 with 64- and 128-bit internal data stream width to our TEC tree implementation. As expected, it shows that Meas-v2 performs clearly better than Meas-v1 in terms of read bandwidth, yielding up to 52 MB/s. Meas-v2 only fetches and decrypts the actually required keys from within intermediate tree nodes and thus allows for faster read access. On the other hand, the write performance is generally lower and only a little better for Meas-v2 than for Meas-v1, because the re-keying step requires to read, modify, and re-encrypt full intermediate nodes in Meas-v2 as well. However, the slightly better write performance of up to 11 MB/s is caused by *ENC* lacking initialization and key derivation in Meas-v2. In terms of the internal data stream width, it shows that despite the 64-bit memory interface, the 128-bit internal interface gives better results for both Meas-v1 and Meas-v2. This is mainly due to the instant availability of the 128-bit keys from caches in the read case, and the faster processing of decrypted keys in the write case.

**Fig. 13:**
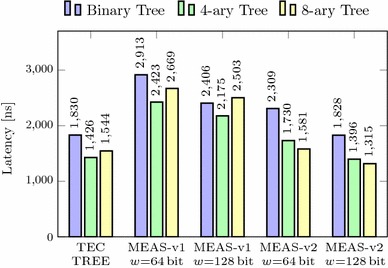
Memory latency determined with LMBENCH (lat_mem_rd 8M)

In terms of tree arity, 4-ary trees give the best write bandwidth for both Meas and the TEC tree. As a closer investigation shows, an arity of four results in the least amount of data being processed when accessing a data block. Regarding read bandwidth, 4-ary trees give the best performance for TEC trees and Meas-v1. However, for Meas-v2 higher arity leads to higher read performance, as Meas-v2 reduces the amount of data to be read from memory during read accesses by providing direct access to the keys within intermediate tree nodes.

#### Latency

Figure 13 shows the latency of all our Meas designs and the TEC tree for different arities. As the main bottleneck of both memory bandwidth and latency is the processing of all the tree nodes, our latency results behave quite similarly to the measured read bandwidth in Fig. 11. In particular, Meas-v2 offers clearly better latency than Meas-v1 across all arities, namely down to 1315 ns (roughly 65 FPGA clock cycles), while the TEC tree behaves quite similarly to Meas-v2. For the TEC tree and Meas-v1, an arity of four yields the lowest latency. However, for Meas-v2 read accesses become faster the higher the arity is. As before, an internal data stream sized 128 bits yields lower latency than 64-bit streams.

**Fig. 14:**
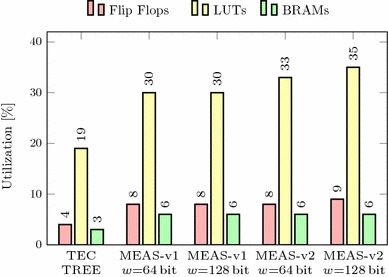
FPGA utilization on XC7Z020 for 8-ary trees

#### Resource utilization

Figure 14 shows the utilization of flip flops, look-up tables (LUTs), and 36 KB block RAMs (BRAM) on the XC7Z020 FPGA SoC for Meas and the TEC tree. In total, this XC7Z020 provides 106,400 flip flops, 53,200 LUTs, and 140 36 KB BRAMs. As the tree arity hardly influences hardware utilization, we focus on the results for 8-ary trees. These results show that all designs are dominated by logic, with an utilization of up to 35 % of LUTs, while the demand for flip flops and BRAMs stays below 10 %. Compared to the TEC tree, Meas consumes 60–80% more logic, because it implements a (tweakable) block cipher and a PRNG in addition to the Ascon permutation.

#### Discussion

Our evaluations indicate quite similar performance of TEC trees and Meas-v2 and higher implementation cost for Meas in general. However, our instances of Meas use 128-bit keys and tags, while our TEC tree implementation operates with smaller 64-bit nonces and tags. Besides, our TEC tree does not offer DPA protection.

On the other hand, TEC trees equipped with protected cryptographic implementations would suffer from significantly lower performance than Meas. Namely, as previous results [43] show, designing masked cryptographic implementations with low latency is difficult, because register stages are needed to ensure side-channel security. For example, the first-order protected, round-parallel Ascon implementation from [24] requires three clock cycles per permutation round. To illustrate the effect of such implementation on the memory bandwidth, we integrated the implementation from [24] into our pipeline from Sect. 8, but omitted any efforts to generate the 320 bits of randomness required per cycle. While this reduced resource utilization by 25% compared to the unprotected, unrolled implementation of Ascon that computes three rounds per cycle, the read and write bandwidth of the TEC tree drop to 2.6 and 0.9 MB/s, respectively. Even when employing Ascon to encrypt and authenticate memory without a tree and replay protection, the bandwidth using the first-order masked implementation merely reached 4.2 MB/s compared to roughly 100 MB/s [58] when using the unprotected implementation.

Summarizing our evaluation results and especially taking into account write performance and side-channel constraints, we conclude that Meas-v2 with arity four is a DPA-secure, highly practical, and hence suitable choice to encrypt RAM. However, as 4-ary Meas-v2 encrypts four 128-bit keys per intermediate tree node with a 64-bit cipher, 4-ary Meas-v2 relies on the assumption of DPA being infeasible given eight different encryptions per key. If DPA on such 8-limiting construction is considered feasible, binary Meas-v2 and 4-ary Meas-v1 are viable alternatives with solid performance results and only four and two encryptions per key, respectively.

## Conclusion

Authentic and encrypted memory is a requirement for storing and processing data in hostile environments where attackers have physical access. The consideration of the imminent threat of side-channel attacks against the involved cryptographic primitives is thus the natural next step.

In this work, we therefore presented Meas, the first Memory Encryption and Authentication Scheme which is secure against DPA attacks. The scheme does not require any DPA-protected primitive, allowing its use in COTS systems. Moreover, Meas provides fast random access on the configured block level and can be adopted for all kinds of use cases including RAM and disk encryption.

The scheme combines the concept of fresh re-keying with authentication trees by storing the involved keys in an encrypted tree structure. While this prevents first-order DPA, masking of the plaintext values flexibly extends the protection of Meas to higher-order DPA if required. Compared to existing schemes, Meas exclusively offers DPA protection by design at roughly the same memory overhead and performance. This is a clear benefit over state-of-the-art memory authentication and encryption techniques, which would face impractical implementation and runtime overheads for DPA-protected implementations if adapted accordingly.

## Appendix A: Authentication trees

In the following we describe three prominent examples of authentication trees, namely Merkle trees [40], Parallelizable Authentication Trees [27] (PAT), and Tamper Evident Counter [18] (TEC) trees. Note, however, that there are also hybrid variants like Bonsai Merkle trees [49], which use elements from both Merkle trees and PATs. The description assumes binary trees, the operator || denotes concatenation.

### A.1 Merkle trees [40]

Merkle trees use a hash function *H* to hash each of the *m* memory blocks $p_i$:

$$\begin{aligned} h_{l, i}&= H(p_i)&0&\le i \le m-1 . \end{aligned}$$

These hashes $h_{l,i}$ are recursively hashed together in a tree structure and the root hash $h_{0,0}$ is put on the secure chip:

$$\begin{aligned} \begin{array}{ll} h_{j, i} = H(h_{j+1,2i} || h_{j+1,2i+1}) &{}\quad 0 \le i \le \frac{m}{2^{l-j}}-1 , \\ &{}\quad 0 \le j \le l-1.\end{array} \end{aligned}$$

### A.2 Parallelizable authentication trees [27]

PATs use a nonce-based MAC and a key *k* to authenticate each of the *m* data blocks $p_i$ using a tag $t_{l,i}$:

$$\begin{aligned} t_{l,i}&= MAC(k; n_{l,i};p_i)&0&\le i \le m-1. \end{aligned}$$

The nonces $n_{l,i}$ are recursively authenticated in a tree structure using again nonce-based MACs. While the key *k* and the root nonce $n_{0,0}$ must be stored on the secure chip, all other nonces and the tags are stored publicly in off-chip memory:

$$\begin{aligned} \begin{array}{ll} t_{j,i} = MAC(k; n_{j,i};n_{j+1,2i} || n_{j+1,2i+1}) &{}\quad 0 \le i \le \frac{m}{2^{l-j}} - 1, \\ &{}\quad 0 \le j \le l-1. \end{array} \end{aligned}$$

### A.3 Tamper evident counter trees [18]

While Merkle trees and PATs provide memory authenticity, TEC trees additionally provide memory confidentiality. Therefore, TEC trees use Added Redundancy Explicit Authenticity [20] (AREA) codes. Hereby, each plain memory block $p_i$ is padded with a nonce $n_{l,i}$ and then encrypted with key *k* using a common block cipher:

$$\begin{aligned} c_{l, i}&= E(k; p_i || n_{l,i})&0&\le i \le m-1. \end{aligned}$$

For verification, a ciphertext $c_{l,i}$ is decrypted to $p'_i || n'_{l,i}$ and $n'_{l,i}$ compared with the original nonce $n_{l,i}$. Hereby, the authenticity is ensured by the diffusion of the block cipher as it makes it hard for the adversary to modify the encrypted nonce $n_{l,i}$. The nonce $n_{l,i}$ is formed from the memory block address and a counter $ctr_{l,i}$ [18]. The nonce counters are recursively authenticated using AREA codes in a tree structure. The key *k* and the root counter $ctr_{0,0}$ are stored on the secure chip:

$$\begin{aligned} \begin{array}{ll} c_{j, i} = E(k; ctr_{j+1,2i} || ctr_{j+1,2i+1} || n_{j, i}) &{}\quad 0 \le i \le \frac{m}{2^{l-j}}-1, \\ &{}\quad 0 \le j \le l-1 . \end{array} \end{aligned}$$

## Footnotes

1 Both implementations are available at https://github.com/IAIK/memsec.
